# Mast Cells, microRNAs and Others: The Role of Translational Research on Colorectal Cancer in the Forthcoming Era of Precision Medicine

**DOI:** 10.3390/jcm9092852

**Published:** 2020-09-03

**Authors:** Giuseppe Sammarco, Gaetano Gallo, Giuseppina Vescio, Arcangelo Picciariello, Gilda De Paola, Mario Trompetto, Giuseppe Currò, Michele Ammendola

**Affiliations:** 1 Department of Health Sciences, University of Catanzaro, Viale Europa, 88100 Catanzaro, Italy; sammarco@unicz.it (G.S.); currog@unicz.it (G.C.); michele.ammendola@unicz.it (M.A.); 2 Department of Medical and Surgical Sciences, University of Catanzaro, Viale Europa, 88100 Catanzaro, Italy; vescio@unicz.it (G.V.); gilda_depaola@libero.it (G.D.P.); 3 Department of Emergency and Organ Transplantation, University “Aldo Moro” of Bari, Piazza G Cesare, 11, 70124 Bari, Italy; arcangelopicciariello@gmail.com; 4 Department of Colorectal Surgery, S. Rita Clinic, 13100 Vercelli, Italy; trompetto.mario@libero.it

**Keywords:** mast cells, microRNAs, KRAS, BRAF, colorectal cancer, precision medicine, translational research

## Abstract

Colorectal cancer (CRC) is a heterogeneous disease, molecularly and anatomically, that develops in a multi-step process requiring the accumulation of several genetic or epigenetic mutations that lead to the gradual transformation of normal mucosa into cancer. In fact, tumorigenesis is extremely complex, with many immunologic and non-immunologic factors present in the tumor microenvironment that can influence tumorigenesis. In the last few years, a role for mast cells (MCs), microRNAs (miRNAs), Kirsten rat sarcoma (KRAS) and v-raf murine sarcoma viral oncogene homologue B (BRAF) in cancer development and progression has been suggested, and numerous efforts have been made to thoroughly assess their correlation with CRC to improve patient survival and quality of life. The identification of easily measurable, non-invasive and cost-effective biomarkers, the so-called “ideal biomarkers”, for CRC screening and treatment remains a high priority. The aim of this review is to discuss the emerging role of mast cells (MCs), microRNAs (miRNAs), KRAS and BRAF as diagnostic and prognostic biomarkers for CRC, evaluating their influence as potential therapy targets in the forthcoming era of precision medicine.

## 1. Introduction

Colorectal cancer (CRC) is a heterogeneous disease [1], molecularly and anatomically [2], that develops in a multi-step process [3] requiring the accumulation of several genetic or epigenetic mutations that lead to the gradual transformation of normal mucosa into cancer.

The global burden of CRC is predicted to increase to more than 2.2 million new cases and 1.1 million fatalities by 2030 [4], and there were 861,700 CRC-related deaths in 2018 [5].

Over 70% of CRC cases are sporadic, 20% of cases have an associated hereditary component, and less than 5% of cases are inherited (Lynch Syndrome, 2–5%).

There are currently three main routes of CRC carcinogenesis: chromosomal instability, DNA replication errors and epigenetic regulation, which includes aberrant hypermethylation and gene silencing [3,6,7]. In fact, recent genome-targeting investigations confirmed that each patient is genetically and epigenetically unique [8]. Nevertheless, regardless of these routes, the tumor microenvironment (TME) [9], a landmark of all solid cancers composed of a variety of cells such as stromal cells, fibroblasts, extracellular matrix (ECM) and vessels, should be considered an influencing factor of CRC development, as already demonstrated by the eminent role of inflammation in patients with inflammatory bowel disease (IBD) [10,11].

CRC becomes symptomatic only at advanced stages [12]; disease curability and highest the chance of patient survival can be achieved only if the disease is detected early with a proper screening method.

In fact, the mortality rate changes totally according to the stage in which the diagnosis is made, and only 80% of patients at primary diagnosis can be potentially cured [13].

Greegor in 1967 described the first attempt at a non-invasive detectable CRC biomarker [14]. However, after thirty years, the attention shifted to molecular biomarkers, i.e., the Kirsten rat sarcoma (KRAS) mutation [15].

Although several non-invasive biomarkers have been proposed so far, the combination of colonoscopy and fecal immunochemical tests (FIT) remains the gold standard for CRC screening.

Unfortunately, participant adherence is crucial, and colonoscopy is invasive, expensive and not free from complications, occurring in between 3% and 16% of cases [16].

In this regard, Computed Tomography (CT) colonography has a higher acceptability rate than colonoscopy [17], but its effectiveness as a screening method is still unclear. Furthermore, FIT has a relatively low sensitivity, depending on the hemoglobin cut-off value, and CT colonography may fail in detecting lesions < 6 mm [18,19,20].

The identification of easily measurable, non-invasive and cost-effective biomarkers, the so-called “ideal biomarkers”, for CRC screening and treatment remains a high priority.

The aim of this review is to discuss the emerging role of mast cells (MCs), microRNAs (miRNAs), KRAS and v-raf murine sarcoma viral oncogene homologue B (BRAF) as diagnostic and prognostic biomarkers for CRC, evaluating their influence as potential therapy targets in the forthcoming era of precision medicine.

## 2. Mast Cells

MCs, from the German *mast* meaning well-fed, were first described by Paul Ehrlich in 1878 [21,22] based on the unique color-changing granules within these cells. In fact, MCs are easily recognizable with characteristic toluidine blue-positive granules in the cytoplasm [22]. Their granules contain several elements, such as histamine, serotonin, heparin, proteases (chymase, tryptase, carboxypeptidase), cytokines and other growth factors (vascular endothelial growth factor, VEGF; fibroblast growth factor-2, FGF-2) [23]. Tryptases and chymases are considered MC-specific proteases, but the granules also contain some non-specific enzymes, such as metalloproteinase-9 [24].

MCs are round or elongated in shape and have diameters ranging from 8 to 20 μm and are bone marrow-derived leukocytes (CD34+) that are not present in avascular tissue [25]; they play a key role in the homeostasis of wound healing as well as in both innate and adaptive immunity. These processes are closely controlled by microenvironmental stimuli, leading to the development of different subtypes.

In contrast to other cells, they leave the bone marrow from an immature non-granulated precursor [26] that circulates in the blood and becomes mature under the influence of growth factors such as stem cell factor (SCF) and the ligand for c-kit receptor (CD117). There are two distinct types of MC: connective tissue and mucosa. Usually, the latter represent 2% of mucosal cells in the healthy gut, but this number can increase in response to different stimuli.

In 1891, an MC was identified by Westphal at the tumor periphery [27]. Since then, several studies have hypothesized the role of MCs in tumor development and progression [28], correlating their infiltration with a poor prognosis in gastrointestinal tumors [29,30,31,32], including CRC [33].

In fact, tumor cells produce SCF, which is the main chemoattractant and survival factor for MCs, with the consequent activation of the c-Kit pathway [34,35,36]. Thus, they are recruited in the initial step of tumorigenesis, promoting the angiogenic switch [37] through the release of anti- or pro-angiogenetic factors based on stimuli received by the TME that can regulate MC and contribute to ECM remodeling, which is crucial for tumor progression, invasion and metastasis. In particular, interleukin (IL)-1, IL-4, IL-6 or Tumor Necrosis Factor alpha (TNF-α) can act as antitumoral factors, whereas FGF-2, VEGF, Matrix Metalloproteinases (MMP-9), tryptase, chymase, IL-8 and IL-10 promote tumorigenesis [38].

Angiogenesis is an indicator of tumor aggressiveness and is essential for tumor spread, providing a vascular route [39]. During tumorigenesis, cancer cells face a shortage of supply of nutrients, which leads to the creation of a hypoxic and acidotic TME. Consequently, angiogenesis can help to restore a sufficient blood supply through the formation of new vessels. In this context, the role of endothelial cells (ECs) is crucial for the ability to suppress recruitment and activity of circulating T cells. Under hypoxic conditions, MCs release IL-6, VEGF-A and C-X-C motif ligand 8 (CXCL8), which promote epithelial-to-mesenchymal transition (EMT) in cancer cells [36].

**Figure 1:**
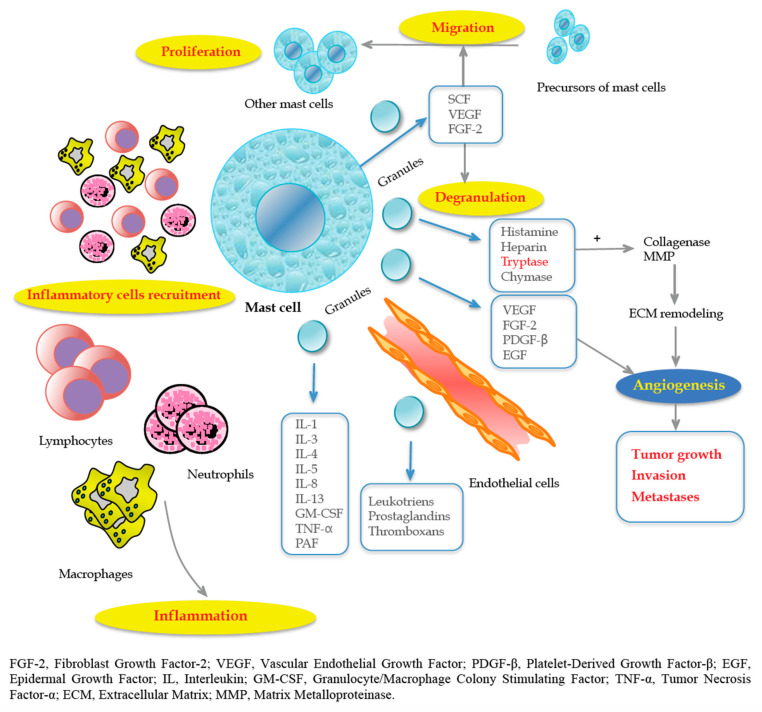
The close relationship between mast cells (MCs) and tumor progression angiogenesis-mediated. Reproduced with permission from Marech et al. [35].

In this respect, according to Engel et al. [40], the number of microvessels is an important predictor of CRC recurrence and time to recurrence. These results were confirmed by Frank et al. [41] in 105 patients with CRC. Furthermore, MCs regulate lymphangiogenesis by releasing VEGF-C and VEGF-D [42,43]. Marech et al. [35] accurately described the biological and translational significance of MCs density in CRC, pointing out the close relationship between MCs and angiogenesis-mediated tumor progression (Figure 1).

Tryptases are tetrameric neutral serine proteases; they are most abundant in MC secretory granules and are the main stimulating factors of neovascularization and EC proliferation [44].

Two main types of MC tryptases exist: alfa and beta. Alpha-tryptases are stored in the granules of MCs, whereas beta-tryptases circulate in the blood as inactive proenzymes and are the major constituents of secretory granules in humans [45].

Positive MCs tryptase expression has a negative effect on prognosis [46], and its density is associated with advanced tumor parameters and aggressive behavior, i.e., tumor size and lymph node metastasis in solid tumors.

Tryptases are agonists of protease-activated receptor-2 (PAR-2), which belongs to the superfamily of G-protein-coupled receptors and is expressed on EC and epithelial cells. Once activated, PAR-2 induces cell proliferation, releasing angiogenic factors and granulocyte-macrophage colony-stimulating factor (GM-CSF).

Histamine is one of the factors released by MCs, and it exerts its functions through four receptors (H1–H4) and has been involved in CRC development. In fact, patients treated with cimetidine (histamine antagonist) reported a significant symptomatic improvement as well as a survival benefit [47,48]. However, the results regarding the role of histamine are still controversial.

High MC density is correlated with advanced stage, tumor progression and poor outcome in CRC [32,49]. Targeted MC therapy is under development, and only a few studies have tried to investigate this issue [50]. Among these studies, tyrosine kinase inhibitors (imatinib and masitinib) for c-Kit receptor and MC tryptase inhibitors (gabexate mesylate, nafamostat mesylate and tranilast) are the best known and most studied [51,52].

Interestingly, in a series of 61 consecutive patients with CRC, the correlation between serum tryptase levels before surgery and tumor-mediated angiogenesis was demonstrated. The authors concluded that elevated levels of serum tryptases after surgery could be indicative of residual tumor tissue or unknown metastases [33]. Ammendola et al. [52] summarized the main in vivo/in vitro strategies of targeted MC therapy in solid tumors and their metastasis. A better understanding of the complex relationships between the whole angiogenesis signaling pathway and MC can represent the future of therapy aimed at arresting tumor growth in gastrointestinal cancer.

## 3. MicroRNA

In the last few years, the field of epigenetics has significantly expanded due to the discovery of new high-throughput microarray-based miRNA profiling platforms such as next-generation sequencing (NGS) that allow genome-wide miRNA and mRNA expression analyses [53]. In this regard, Schetter et al. [54] published the first systemic and comprehensive array-based analysis to evaluate 389 miRNA expression levels in CRC and normal colonic tissues, identifying 37 aberrantly expressed miRNAs in CRC and demonstrating the overexpression of miRNA-20a, miRNA-21, miRNA-106a, miRNA-181b, and miRNA-203 in tumor tissues.

miRNAs are a class of short endogenous single-stranded non-coding RNAs that are 18–25 nucleotides in length and are able to post-transcriptionally repress gene expression by binding to the 3′ untranslated region (UTR) of their target mRNAs [7,55].

It was first discovered in 1993 as a developmental regulator of the nematode Caenorhadbitis elegans [56,57]. Since the first abnormalities of lin-4 and let-7 loss of function were observed, the number of publications regarding miRNAs has expanded enormously.

**Figure 2:**
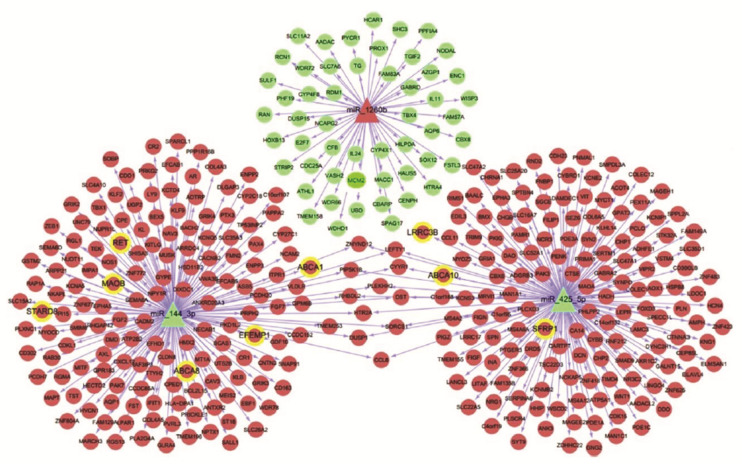
Schematic of the selected miRNA–mRNA networks involved in colorectal cancer (CRC). Red indicates a higher level in CRC and green represents a lower level compared with controls. The triangle refers to miRNAs, and the circle refers to target genes. Yellow refers to MARKER genes related to CRC that are recruited in the Comparative Toxicogenomics Database (CTD) database. In tumors, miR-1260b possesses 53 target genes that are significantly down-regulated, whereas miR-425-5p and miR-144-3p possess 159 and 162 target genes, respectively, that were significantly upregulated. A total of nine MARKER-related genes are found in CRC, including ATP-binding Cassette Transporter A1 (ABCA1), ATP-binding Cassette Transporter A8 (ABCA8), EGF Containing Fibulin Extracellular Matrix Protein 1 (EFEMP1), Monoamine Oxidase B (MAOB), REarranged during Transfection (RET), StAR Related Lipid Transfer Domain Containing 8 (STARD8), ATP-binding Cassette Transporter A10 (ABCA10), Leucine Rich Repeat Containing 3B (LRRC3B), and Secreted Frizzled Related Protein 1 (SFRP1). Reproduced with permission from Tan et al. [61].

The miRNA formation process begins with synthesis into the nucleus by RNA polymerase II of a primary miRNA (pri-miRNA), a 5′-cap long transcript of an approximately 70-nucleotide hairpin structure and 3′ poly(A) tail. The latter is then processed to pre-miRNA by RNase III enzyme Drosha and its cofactor DGCR8 with the removal of both the 5′-cap and 3′-poly(A) tail. The pre-miRNA is transported to the cytoplasm by exportin 5 and processed into a mature miRNA and its complementary miRNA strand by the ribonuclease III enzyme Dicer [58]. After all these processes, the mature miRNA is integrated into the RNA inducing silencing complex (RISC) and negatively regulates the expression of several target mRNAs, leading to their degradation or translational inhibition [59,60]. A single miRNA is able to regulate up to hundreds of target mRNAs [61] (Figure 2).

In 2002, Calin et al. [62] first reported the role of miRNAs in cancer, demonstrating the reduction of miRNA-15 and miRNA-16 in patients with chronic lymphocytic leukemia.

miRNAs have been found in different body fluids, such as serum, plasma, urine, saliva, and tissues, where they are stable [7].

There are several studies that report the role of miRNAs in a variety of biological processes and immune responses as well as the correlation between dysregulated miRNAs and the aberrant regulation of signaling pathways, including angiogenesis, drug resistance and EMT [63,64,65]; miRNAs play these roles by regulating oncogene and tumor suppressor gene expression [66,67].

miRNAs can circulate freely or via exosomes surviving in a stable form [68]. They are well protected from endogenous degradation and are reproducible and consistent plasma markers [69].

Chen and colleagues [70], in their study of serum miRNA profiling in CRC patients, first declared the high degree of miRNA CRC specificity by demonstrating the unique expression of 14 miRNAs in CRC patients. None of the 69 serum miRNAs identified were expressed in healthy volunteers.

miRNA-21 is one of the most promising and well-known oncogenic miRNAs correlating with all stages of tumorigenesis [7]. It is differentially expressed in both adenoma and CRC compared to their normal counterparts, revealing a possible distinguishing feature between cancer and normal samples [71,72,73] and allowing for an early diagnosis. The latter concept is key for improving the survival of patients with CRC, and further studies reporting an upregulation of miRNAs in adenoma tissues compared with normal healthy mucosa confirmed the trend [74]. The plasma levels of three miRNAs, miR-24, miR-320a and miR423-5p, can be monitored to predict the post-operative risk of metastasis in CRC patients [75].

Fecal miRNAs have been shown to correlate with tumor stage [76,77], probably because they are continuously released into the intestinal lumen by CRC cells and can be detected in stool samples [78]. Stool comes into direct contact with the intestinal lumen, providing a rationale for the use of miRNAs as biomarkers in CRC [79].

Koga et al. [80] studied exfoliated colonocytes by comparing miRNAs expression in 197 patients with CRC and 119 healthy controls. The authors demonstrated that the miRNA-17-92 cluster and miRNA-135 were most highly expressed in patients with CRC (*p* < 0.0001). These results were confirmed by Wu et al. [81] and Link et al. [82]. Many other authors support the downregulation or overexpression of miRNAs in the fecal samples of CRC patients compared to those of healthy controls [83].

Regarding miRNAs in tumor tissue, miRNA-143 and miRNA-145 were the first to be associated with CRC tumorigenesis. In particular, both of them were downregulated in precancerous and CRC tissues compared with normal healthy mucosa [84]. Subsequent studies have shown their role as tumor suppressors [85,86]. MiRNAs contained in exosomes can activate Toll-like-receptor 8 with the release of IL-6 and TNF-α, increasing tumor growth [87].

**Figure 3:**
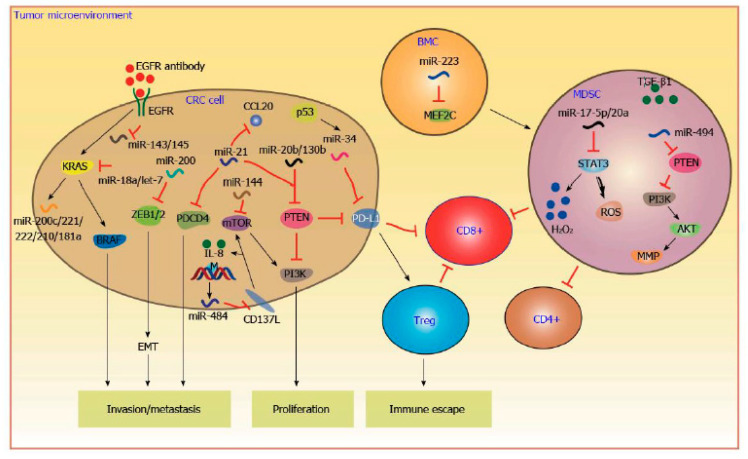
MicroRNAs control colorectal carcinoma progression through modulating anti-tumor immune responses in the tumor microenvironment. For example, miR-20b and miR-130b repress the expression of Phosphatase and tensin homolog (PTEN) and increase the activity of Phosphoinositide 3-kinase (PI3K), leading to the increased viability of CRC cells. MiR-21 promotes CRC invasion and metastasis by directly targeting Programmed Cell Death 4 (PDCD4). Furthermore, miR-34a enhances cluster of differentiation 8 (CD8+) T cells cytotoxicity by repressing the expression of programmed death-ligand 1 (PD-L1), contributing to the elimination of CRC cells by cytotoxic T lymphocytes (CTLs). Reproduced with permission from [68].

The development of miRNA-targeted therapy is promising considering their crucial role in CRC tumorigenesis (Figure 3). In fact, some miRNAs, such as miRNA-320 and miRNA-498, were significantly correlated with recurrence-free survival in colon cancer [88]. Furthermore, increased levels of miRNA-21 in tumor tissues have been associated with poor recurrence-free cancer-specific survival [7].

Recently, some studies have shown the interaction of miRNAs with other biomarkers, such as KRAS [89,90], and their consequent role in predicting treatment response to classical cytotoxic chemotherapy or targeted therapy (anti-epidermal growth factor receptor (EGFR) and anti-VEGF) [91,92,93,94].

Patients with CRC and high levels of let-7 may benefit from anti-EGFR therapy (cetuximab) due to its downregulatory effect on KRAS [95]. Valeri et al. [96] demonstrated the role of miRNA-21 in conferring resistance to 5- fluorouracil (FU) downregulating MutS homologue 2 (MSH-2). An inhibitor of this miRNA is under evaluation for the treatment of hepatocellular carcinoma [97]. High levels of miRNA-203 have been correlated with oxaliplatin resistance [93], and the deregulation of other miRNAs, such as miRNA-10b [98], is associated with 5-fluorouracil (FU) and irinotecan resistance [99].

## 4. KRAS

Rat sarcoma (RAS) proteins are a group of nucleotide guanosine triphosphate (GTPases) involved in several crucial cell processes, and their mutations play a pivotal role in human carcinogenesis [100].

Activating mutations in the RAS genes occur in approximately 45% of CRC. Among them, the KRAS mutation is mainly involved in preventing the hydrolysis of the GTP complex, leading to cell proliferation via several signaling pathways [101,102,103]. The proto-oncogene KRAS recruits BRAF, initiating mitogen-activated protein kinase (MAPK) signaling and leading to the expression of multiple downstream effectors involved in cell proliferation, differentiation, and survival [104].

The KRAS gene contains six exons, and the most frequent mutations occur at codons 12 and 13. The results of studies performed on patients with advanced or recurrent CRC have revealed that out of 35% of patients with KRAS mutations, 25% had mutations at codon 12, and 10% had mutations at codon 13 [105].

Constitutively activated KRAS has several effects on tumor initiation and clinicopathological features, influencing tumor progression, local invasion, and metastasis formation [106].

The role of KRAS in facilitating tumor metastasis depends on the effect of activated metalloproteases-7 and urokinase plasminogen activator, which breakdown and promote migration through the basement membrane, leading to the dissemination of cancer cells from the primary tumor [107,108,109].

Although there is no clear evidence of the relationship between KRAS mutations and CRC stage and location, patients with codon 12 mutations were found to be more commonly affected by advanced stage proximal tumors with mucinous histology [110].

Furthermore, some authors showed that codon 13 mutation was related to a 40% increase in short-term mortality from colon cancer and was associated with advanced Dukes stage [110,111,112].

However, even if there is some evidence suggesting that the KRAS-mutated tumors have more aggressive biologic behavior, the effect of the mutational status on clinical outcomes is under discussion and still not well defined.

For the understanding of the molecular mechanisms by which the mutational status affects CRC growth and metastasis, KRAS mutational analysis has become an important step in the era of personalized medicine [113].

The association between KRAS mutation and patient survival remains contended and further studies are required to explore the prognostic value of KRAS status in left-sided and right-sided CRC. In fact, the prognostic value of KRAS status could also depend on the location of the primary tumor and left-sided CRC patients have a greater risk of death [114,115].

The prognostic role of KRAS mutations is controversial, but a recent systematic review showed a negative effect in the adjuvant and metastatic setting. Actually, in the case of liver metastasis from CRC, the RAS mutation has been reported to be the most common negative prognostic factor for overall survival (OS) [116]. Furthermore, KRAS mutations seem to be associated with worse survival among patients undergoing repeat hepatectomy [117].

KRAS mutational analysis should be carried out in all metastatic CRC patients at the beginning of treatment aiming to tailor their targeted therapy [112].

The rationale beyond the accurate study of the specific type of KRAS mutation can be explained by the fact that the constitutively activated RAS/RAF/MAPK pathway (in case of mutation in codons 12 and 13) makes the targeting of epidermal growth factor receptor (EGFR) therapeutically useless.

Therefore, mutated KRAS represents a negative predictive biomarker for the response to anti-EGFR therapy in patients affected by metastatic CRC [118].

Recently, blood-based genomic profiling (“liquid biopsy”) has been demonstrated to be a useful method to study and monitor tumor genomes, allowing the detection of early signs of disease progression even months before clinical and radiological confirmation [119].

In fact, the analysis of circulating cell-free tumor DNA can be used to qualify and quantify genomic imbalances and can provide promising biomarkers for personalizing therapy [120].

The emerging field of liquid biopsies to detect mutational status could have meaningful clinical implications to individualize an optimal targeted therapy, especially for patients with KRAS-mutated metastatic CRC [121].

## 5. BRAF

Several pathways are involved in CRC carcinogenesis, and phenotypic and molecular comprehensive characterization represents an important key step in defining diagnosis, prognosis and treatment [122,123]. Cell proliferation, differentiation, migration and survival are driven by the MAPK cascade, which includes the RAS small guanidine triphosphate that leads to the activation of RAF family proteins. RAF proteins cause activation, through phosphorylation, of the extracellular signal-regulated kinase and have an effect on multiple transcription factors and on the regulation of cellular processes [124]. The whole signaling cascade is well known as RAS/RAF/ Mitogen-activated protein kinase (MEK)/ extracellular signal-regulated kinase (ERK) [125].

The role of BRAF (v-raf murine sarcoma viral oncogene homologue B) is to activate the MAPK pathway, which regulates cell growth, proliferation, and differentiation, and to influence other key cellular processes, including cell migration (through RHO small GTPases), apoptosis [through B-cell lymphoma 2 (BCL-2)], and survival (through the HIPPO pathway) [126,127].

Although BRAF can be mutated in several sites, the V600E mutation occurs in approximately 80% of cases and is characterized by a nucleotide (1799T > A) change resulting in an amino acid change leading to constitutive kinase activity [128].

The percentage of BRAF gene mutations has been reported in 15% of all human cancer types and in approximately 10% of CRC [6].

The study of BRAF gene mutations has individualized two subsets of localized and metastatic CRC characterized by specific clinicopathological and molecular features [129,130,131].

CRC with mutated BRAF are characterized by aggressive behavior with frequent peritoneal involvement (distinct pattern of metastatic spread) and high resistance to standard targeted therapy [132,133]. V600E BRAF mutation has been shown to be an acquired genetic event, occurring more frequently in older women (>60 years old) with right-sided primary tumors, CRC stage II/III and metastatic disease [130,134,135].

Conversely, patients with non-V600E mutations are usually younger, with left-sided primary tumors in a less advanced stage. Serrated polyps are considered precursor lesions and often exhibit an extensive DNA methylation process that may occur in the MutL homolog 1 (MLH1) promoter (a gene of the mismatch repair system), leading to ‘sporadic’ microsatellite instability (MSI) in 60% of BRAF-mutated CRCs [136,137].

The deficiency of the mismatch repair machinery due to MSI has been considered to be a prognostic factor; in fact, when compared to CRC “stable” BRAF-mutated patients, the “instable” (MSI) forms have been reported to have a better prognosis [138].

Microsatellite “stable” BRAF-mutated CRCs share some features with MSI and BRAF-wild-type forms, such as proximal colon location and mucin production, but they usually affect younger patients with no difference in gender and show more aggressive morphological behavior, such as frequent tumor budding, lymphatic, perineural, and vascular invasion and early lymph node metastases [139].

With regard to the prognostic impact of BRAF-mutated CRC, although new chemotherapeutic regimens and targeted drugs (such as anti-epidermal growth factor receptor-EGFR monoclonal antibodies) have been proposed and Food and Drug Administration FDA-approved [140,141], the overall survival (OS) remains poor and depends on the type of the mutation and the MSI status [142,143]. Recently, Loupakis et al. proposed a “complete prognostic score” able to stratify patients into three groups (low, intermediate and high risk) with different outcomes [144].

Although several studies have been conducted with the aim of defining the role of BRAF mutations as potential biomarkers, questions regarding their predictive value are still debated, opening new frontiers of targeted therapies.

A study on CRC patients with BRAF mutations [145] limited by a small sample size suggests a potential negative predictive role of BRAF mutations in patients treated with anti-EGFR drugs. Nevertheless, anti-EGFR drugs associated with FOLFOXIRI (FOLinic acid, Fluorouracil, Oxaliplatin, IRInotecan) seem to result in a higher objective response rate (ORR) than FOLFOXIRI alone in patients with the V600E mutation [146]. BRAF inhibitors have revolutionized the treatment of V600E BRAF metastatic melanoma [147], but the rationale underlying the use of such inhibitors cannot explain a response in CRC patients because the molecular landscape is more complex and heterogeneous [148].

Recently, in the BEACON trial [149], an open-label, phase III trial, it has been demonstrated that a combination of encorafenib, cetuximab and binimetinib (triplet-therapy group) resulted in a higher response rate and higher overall survival in patients with metastatic CRC with the V600E BRAF mutations if compared with the standard therapy, i.e., cetuximab and irinotecan or cetuximab and FOLFIRI.

In the era of precision medicine, the predictive value of BRAF mutation in CRCs is still controversial and needs more efforts to further stratify the BRAF mutant population (considering non-V600E BRAF mutations) and to include other potential targets, improving the efficacy of multiple personalized therapies and establishing a new standard of care.

## 6. Conclusions

CRC remains one of the leading causes of death worldwide, although several screening tests have been described so far.

Currently, there is not yet a clear recommendation about the clinical use of biomarkers in CRC but the understanding of their functions and role in screening, diagnosis, treatment and prognosis and the consequent development of precision medicine is crucial for changing the outcomes of patients with CRC in the next few years.

## References

[1] Molinari C., Marisi G., Passardi A., Matteucci L., De Maio G., Ulivi P. (2018). Heterogeneity in Colorectal Cancer: A Challenge for Personalized Medicine?. Int. J. Mol. Sci..

[2] Alvaro E., Cano J.M., Garcia J.L., Brandariz L., Olmedillas-Lopez S., Arriba M., Rueda D., Rodriguez Y., Canete A., Arribas J. (2019). Clinical and Molecular Comparative Study of Colorectal Cancer Based on Age-of-Onset and Tumor Location: Two Main Criteria for Subclassifying Colorectal Cancer. Int. J. Mol. Sci..

[3] Fearon E.R., Vogelstein B. (1990). A genetic model for colorectal tumorigenesis. Cell.

[4] Arnold M., Sierra M.S., Laversanne M., Soerjomataram I., Jemal A., Bray F. (2017). Global patterns and trends in colorectal cancer incidence and mortality. Gut.

[5] Ferlay J., Colombet M., Soerjomataram I., Mathers C., Parkin D.M., Piñeros M., Znaor A., Bray F. (2019). Estimating the global cancer incidence and mortality in 2018: GLOBOCAN Sources and Methods. Int. J. Cancer.

[6] Gallo G., Sena G., Vescio G., Papandrea M., Sacco R., Trompetto M., Sammarco G. (2019). The prognostic value of KRAS and BRAF in stage I-III colorectal cancer. A systematic review. Ann. Ital. Chir..

[7] Pellino G., Gallo G., Pallante P., Capasso R., De Stefano A., Maretto I., Malapelle U., Shengyang Q., Nikolaou S., Barina A. (2018). Noninvasive Biomarkers of Colorectal Cancer: Role in Diagnosis and Personalised Treatment Perspectives. Gastroenterol. Res. Pract..

[8] Carethers J.M., Jung B.H. (2015). Genetics and Genetic Biomarkers in Sporadic Colorectal Cancer. Gastroenterology.

[9] Hui L., Chen Y. (2015). Tumor Microenvironment: Sanctuary of the Devil. Cancer Lett..

[10] Gallo G., Kotze P.G., Spinelli A. (2018). Surgery in ulcerative colitis: When? How?. Best Pract. Res. Clin. Gastroenterol..

[11] Pellino G., Marcellinaro R., Candilio G., De Fatico G.S., Guadagno E., Campione S., Santangelo G., Reginelli A., Sciaudone G., Riegler G. (2016). The Experience of a Referral Centre and Literature Overview of GIST and Carcinoid Tumours in Inflammatory Bowel Diseases. Int. J. Surg..

[12] Wisselink D.D., Braakhuis L.L.F., Gallo G., van Grevenstein W.M.U., van Dieren S., Kok N.F.M., de Reuver P.R., Tanis P.J., de Hingh I.H.J.T. (2019). Systematic review of published literature on Oxaliplatin and Mitomycin C as chemotherapeutic agents for Hyperthermic Intraperitoneal Chemotherapy in patients with peritoneal metastases from colorectal cancer. Crit. Rev. Oncol. Hematol..

[13] Rawson J.B., Bapat B. (2012). Epigenetic Biomarkers in Colorectal Cancer Diagnostics. Expert Rev. Mol. Diagn..

[14] Greegor D.H. (1967). Diagnosis of Large-Bowel Cancer in the Asymptomatic Patient. JAMA.

[15] Sidransky D., Tokino T., Hamilton S.R., Kinzler K.W., Levin B., Frost P., Vogelstein B. (1992). Identification of Ras oncogene mutations in the stool of patients with curable colorectal tumors. Science.

[16] Von Karsa L., Patnick J., Segnan N., Atkin W., Halloran S., Lansdorp-Vogelaar I., Malila N., Minozzi S., Moss S., Quirke P. (2013). European Guidelines for Quality Assurance in Colorectal Cancer Screening and Diagnosis: Overview and Introduction to the Full Supplement Publication. Endoscopy.

[17] Rosman A.S., Korsten M.A. (2007). Meta-analysis comparing CT colonography, air contrast barium enema, and colonoscopy. Am. J. Med..

[18] Rockey D.C., Paulson E., Niedzwiecki D., Davis W., Bosworth H.B., Sanders L., Yee J., Henderson J., Hatten P., Burdick S. (2005). Analysis of air contrast barium enema, computed tomographic colonography, and colonoscopy: Prospective comparison. Lancet.

[19] Graser A., Stieber P., Nagel D., Schäfer C., Horst D., Becker C.R., Nikolaou K., Lottes A., Geisbüsch S., Kramer H. (2009). Comparison of CT colonography, colonoscopy, sigmoidoscopy and faecal occult blood tests for the detection of advanced adenoma in an average risk population. Gut.

[20] Gallo G. (2020). Preoperative Colorectal-Cancer Detection: Do We Need Anything Else? An Invited Brief Commentary on Is CT Scan More Accurate than Endoscopy in Identifying Distance from the Anal Verge for Left-sided Colon Cancer? A Comparative Cohort Analysis. J. Investig. Surg..

[21] Ehrlich P. (2013). Beiträge zur Theorie und Praxis der Histologischen Färbung. The Collected Papers of Paul Ehrlich.

[22] Crivellato E., Beltrami C.A., Mallardi F., Ribatti D. (2003). Paul Ehrlich’s doctoral thesis: A milestone in the study of mast cells. Br. J. Haematol..

[23] Blank U. (2011). The mechanism of exocytosis in Mast Cells. Adv. Exp. Med. Biol..

[24] Wernersson S., Pejler G. (2014). Mast cell secretory granules: Armed for battle. Nat. Rev. Immunol..

[25] Silver R., Silverman A.J., Vitkovic L., Lederhendler I.I. (1996). Mast cells in the brain: Evidence and functional significance. Trends. Neurosci..

[26] Okayama Y., Kawakami T. (2006). Development, Migration, and Survival of Mast Cells. Immunol. Res..

[27] Westphal E., Ehrlich E. (1891). Uber Mastzellen. Farbenanalytische Untersuchungen.

[28] Conti P., Castellani M.L., Kempuraj D., Salini V., Vecchiet J., Tetè S., Mastrangelo F., Perrella A., De Lutiis M.A., Tagen M. (2007). Role of mast cells in tumor growth. Ann. Clin. Lab. Sci..

[29] Acikalin M.F., Oner U., Topcu I., Yasar B., Kiper H., Colak E. (2005). Tumour angiogenesis and mast cell density in the prognostic assessment of colorectal carcinomas. Dig. Liver Dis..

[30] Melillo R.M., Guarino V., Avilla E., Galdiero M.R., Liotti F., Prevete N., Rossi F.W., Basolo F., Ugolini C., de Paulis A. (2010). Mast cells have a protumorigenic role in human thyroid cancer. Oncogene.

[31] Ammendola M., Sacco R., Donato G., Zuccala V., Russo E., Luposella M., Vescio G., Rizzuto A., Patruno R., De Sarro G. (2013). Mast cell positivity to tryptase correlates with metastatic lymph nodes in gastrointestinal cancer patients treated surgically. Oncology.

[32] Malfettone A., Silvestris N., Saponaro C., Ranieri G., Russo A., Caruso S., Popescu O., Simone G., Paradiso A., Mangia A. (2013). High density of tryptase-positive mast cells in human colorectal cancer: A poor prognostic factor related to protease-activated receptor 2 expression. J. Cell. Mol. Med..

[33] Ammendola M., Rosario S., Giuseppe S., Giuseppe D., Montemurro S., Ruggieri E., Patruno R., Marech I., Cariello M., Vacca A. (2014). Correlation between serum tryptase, mast cells positive to tryptase and microvascular density in colo-rectal cancer patients: Possible biological-clinical significance. PLoS ONE.

[34] Li G., Yang S., Shen P., Wu B., Sun T., Sun H., Ji F., Zhou D. (2018). SCF/c-KIT signaling promotes mucus secretion of colonic goblet cells and development of mucinous colorectal adenocarcinoma. Am. J. Cancer Res..

[35] Marech I., Ammendola M., Gadaleta C., Zizzo N., Oakley C., Gadaleta C.D., Ranieri G. (2014). Possible biological and translational significance of mast cells density in colorectal cancer. World J. Gastroenterol..

[36] Yu Y., Blokhuis B., Derks Y., Kumari S., Garssen J., Redegeld F. (2018). Human mast cells promote colon cancer growth via bidirectional crosstalk: Studies in 2D and 3D coculture models. Oncoimmunology.

[37] Coussens L.M., Raymond W.W., Bergers G., Laig-Webster M., Behrendtsen O., Werb Z., Caughey G.H., Hanahan D. (1999). Inflammatory mast cells up-regulate angiogenesis during squamous epithelial carcinogenesis. Genes Dev..

[38] Cimpean A.M., Tamma R., Ruggieri S., Nico B., Toma A., Ribatti D. (2017). Mast cells in breast cancer angiogenesis. Crit. Rev. Oncol. Hematol..

[39] Folkman J. (1990). What is the evidence that tumors are angiogenesis dependent?. J. Natl. Cancer Inst..

[40] Engel C.J., Bennett S.T., Chambers A.F., Doig G.S., Kerkvliet N., O’Malley F.P. (1996). Tumor angiogenesis predicts recurrence in invasive colorectal cancer when controlled for Dukes staging. Am. J. Surg. Pathol..

[41] Frank R.E., Saclarides T.J., Leurgans S., Speziale N.J., Drab E.A., Rubin D.B. (1995). Tumor angiogenesis as a predictor of recurrence and survival in patients with node-negative colon cancer. Ann. Surg..

[42] Detoraki A., Staiano R.I., Granata F., Giannattasio G., Prevete N., de Paulis A., Ribatti D., Genovese A., Triggiani M., Marone G. (2009). Vascular endothelial growth factors synthesized by human lung mast cells exert angiogenic effects. J. Allergy Clin. Immunol..

[43] Sammarco G., Varricchi G., Ferraro V., Ammendola M., De Fazio M., Altomare D.F., Luposella M., Maltese L., Currò G., Marone G. (2019). Mast Cells, Angiogenesis and Lymphangiogenesis in Human Gastric Cancer. Int. J. Mol. Sci..

[44] Guo X., Zhai L., Xue R., Shi J., Zeng Q., Gao C. (2016). Mast cell tryptase contributes to pancreatic cancer growth through promoting angiogenesis via activation of angiopoietin-1. Int. J. Mol. Sci..

[45] Payne V., Kam P.C.A. (2004). Mast cell tryptase: A review of its physiology and clinical significance. Anaesthesia.

[46] Marichal T., Tsai M., Galli S.J. (2013). Mast cells: Potential positive and negative roles in tumor biology. Cancer Immunol. Res..

[47] Adams W.J., Morris D.L. (1994). Short-course cimetidine and survival with colorectal cancer. Lancet.

[48] Matsumoto S. (1995). Cimetidine and survival with colorectal cancer. Lancet.

[49] Gulubova M., Vlaykova T. (2009). Prognostic significance of mast cell number and microvascular density for the survival of patients with primary colorectal cancer. J. Gastroenterol. Hepatol..

[50] Ammendola M., Zuccalà V., Patruno R., Russo E., Luposella M., Amorosi A., Vescio G., Sammarco G., Montemurro S., De Sarro G. (2013). Tryptase-positive mast cells and angiogenesis in keloids: A new possible post-surgical target for prevention. Updates Surg..

[51] Patruno R., Marech I., Zizzo N., Ammendola M., Nardulli P., Gadaleta C., Introna M., Capriuolo G., Rubini R.A., Ribatti D. (2014). c-Kit expression, angiogenesis, and grading in canine mast cell tumour: A unique model to study c-Kit driven human malignancies. BioMed. Res. Int..

[52] Ammendola M., Sacco R., Sammarco G., Luposella M., Patruno R., Gadaleta C.D., De Sarro G., Ranieri G. (2016). Mast Cell-Targeted Strategies in Cancer Therapy. Transfus. Med. Hemother..

[53] Ferrero G., Cordero F., Tarallo S., Arigoni M., Riccardo F., Gallo G., Ronco G., Allasia M., Kulkarni N., Matullo G. (2017). Small non-coding RNA profiling in human biofluids and surrogate tissues from healthy individuals: Description of the diverse and most represented species. Oncotarget.

[54] Schetter A.J., Leung S.Y., Sohn J.J., Zanetti K.A., Bowman E.D., Yanaihara N., Yuen A.T., Chan T.L., Kwong D.L.W., Au G.K.H. (2008). MicroRNA expression profiles associated with prognosis and therapeutic outcome in colon adenocarcinoma. JAMA.

[55] Fabbri M., Calin G.A. (2010). Epigenetics and miRNAs in human cancer. Adv. Genet..

[56] Lee R.C., Feinbaum R.L., Ambros V. (1993). The C. Elegans Heterochronic Gene lin-4 Encodes Small RNAs with Antisense Complementarity to lin-14. Cell.

[57] Wightman B., Ha I., Ruvkun G. (1993). Posttranscriptional Regulation of the Heterochronic Gene lin-14 by lin-4 Mediates Temporal Pattern Formation in C. Elegans. Cell.

[58] Esquela-Kerscher A., Slack F.J. (2006). Oncomirs-microRNAs with a role in cancer. Nat. Rev. Cancer.

[59] Ambros V., Bartel B., Bartel D.P., Burge C.B., Carrington J.C., Chen X., Dreyfuss G., Eddy S.R., Griffiths-Jones S., Mashall M. (2003). A uniform system for microRNA annotation. RNA.

[60] Mendell J.T., Olson E.N. (2012). MicroRNAs in stress signaling and human disease. Cell.

[61] Tan Y., Lin J.J., Yang X., Gou D.M., Fu L., Li F.R., Yu X.F. (2019). A panel of three plasma microRNAs for colorectal cancer diagnosis. Cancer Epidemiol..

[62] Calin G.A., Dumitru C.D., Shimizu M., Bichi R., Zupo S., Noch E., Aldler H., Rattan S., Keating M., Rai K. (2002). Frequent deletions and down-regulation of micro-RNA genes miR15 and miR16 at 13q14 in chronic lymphocytic leukemia. Proc. Natl. Acad. Sci. USA.

[63] Poursheikhani A., Abbaszadegan M.R., Nokhandani N., Kerachian M.A. (2020). Integration analysis of long non-coding RNA (lncRNA) role in tumorigenesis of colon adenocarcinoma. BMC Med. Genom..

[64] Sipos F., Galamb O. (2012). Epithelial-to-mesenchymal and mesenchymal-toepithelial transitions in the colon. World J. Gastroenterol..

[65] Fish J.E., Santoro M.M., Morton S.U., Yu S., Yeh R.F., Wythe J.D., Ivey K.N., Bruneau B.G., Stainer D.Y., Srivastava D. (2008). miR-126 regulates angiogenic signaling and vascular integrity. Dev. Cell.

[66] Calin G.A., Croce C.M. (2006). MicroRNA-cancer connection: The beginning of a new tale. Cancer Res..

[67] Chauhan N., Dasmana A., Jaggi M., Chauhan S.C., Yallapu M.M. (2020). miR-205: A Potential Biomedicine for Cancer Therapy. Cells.

[68] Li X., Nie J., Mei Q., Han W.D. (2016). MicroRNAs: Novel immunotherapeutic targets in colorectal carcinoma. World J. Gastroenterol..

[69] Mitchell P.S., Parkin R.K., Kroh E.M., Fritz B.R., Wyman S.K., Pogosova-Agadjanyan E.L., Peterson A., Noteboom J., O’Briant K.C., Allen A. (2008). Circulating microRNAs as stable blood-based markers for cancer detection. Proc. Natl. Acad. Sci. USA.

[70] Chen X., Ba Y., Ma L., Cai X., Yin Y., Wang K., Guo J., Zhang Y., Chen J., Guo X. (2008). Characterization of microRNAs in serum: A novel class of biomarkers for diagnosis of cancer and other diseases. Cell Res..

[71] Hollis M., Nair K., Vyas A., Chaturvedi L.S., Gambhir S., Vyas D. (2015). MicroRNAs potential utility in colon cancer: Early detection, prognosis, and chemosensitivity. World J. Gastroenterol..

[72] Ghareib A.F., Mohamed R.H., Abd El-Fatah A.R., Saadawy S.F. (2020). Assessment of Serum MicroRNA-21 Gene Expression for Diagnosis and Prognosis of Colorectal Cancer. J. Gastrointest. Cancer.

[73] Thomas A.M., Manghi P., Asnicar F., Pasolli E., Armanini F., Moreno Z., Beghini F., Manara S., Karcher N., Pozzi C. (2019). Metagenomic analysis of colorectal cancer datasets identifies cross-cohort microbial diagnostic signatures and a link with choline degradation. Nat. Med..

[74] Nagel R., le Sage C., Diosdado B., van der Waal M., Oude Vrielink J.A.F., Brolijn A., Meijer G.A., Agami R. (2008). Regulation of the adenomatous polyposis coli gene by the miR-135 family in colorectal cancer. Cancer Res..

[75] Fang Z., Tang J., Bai Y., Lin H., You H., Jin H., Lin L., You P., Li J., Dai Z. (2015). Plasma levels of microRNA-24, microRNA-320a, and microRNA-423-5p are potential biomarkers for colorectal carcinoma. J. Exp. Clin. Cancer Res..

[76] Liu S., Weiner H.L. (2016). Control of the gut microbiome by fecal microRNA. Microb. Cell.

[77] Ahmed F.E., Ahmed N.C., Vos P.W., Bonnerup C., Atkins J.N., Casey M., Nuovo G.J., Naziri W., Wiley J.E., Mota H. (2013). Diagnostic microRNA markers to screen for sporadic human colon cancer in stool: I. Proof of principle. Cancer Genom. Proteom..

[78] Ahlquist D.A. (2010). Molecular detection of colorectal neoplasia. Gastroenterology.

[79] Tarallo S., Ferrero G., Gallo G., Francavilla A., Clerico G., Realis Luc A., Manghi P., Thomas A.M., Vineis P., Segata N. (2019). Altered Fecal Small RNA Profiles in Colorectal Cancer Reflect Gut Microbiome Composition in Stool Samples. Msystems.

[80] Koga Y., Yasunaga M., Takahashi A., Kuroda J., Moriya Y., Akasu T., Fujita S., Yamamoto S., Baba H., Matsumura Y. (2010). MicroRNA expression profiling of exfoliated colonocytes isolated from feces for colorectal cancer screening. Cancer Prev. Res..

[81] Wu C.W., Ng S.S., Dong Y.J., Ng S.C., Leung W.W., Lee C.W., Wong Y.N., Chan F.K.L., Sung J.J.Y. (2012). Detection of miR-92a and miR-21 in stool samples as potential screening biomarkers for colorectal cancer and polyps. Gut.

[82] Link A., Balaguer F., Shen Y., Nagasaka T., Lozano J.J., Boland C.R., Goel A. (2010). Fecal MicroRNAs as novel biomarkers for colon cancer screening. Cancer Epidemiol. Biomark. Prev..

[83] Sarshar M., Scribano D., Ambrosi C., Palamara A.T., Masotti A. (2020). Fecal microRNAs as Innovative Biomarkers of Intestinal Diseases and Effective Players in Host-Microbiome Interactions. Cancers.

[84] Michael M.Z., O’Connor S.M., van Holst Pellekaan N.G., Young G.P., James R.J. (2003). Reduced accumulation of specific microRNAs in colorectal neoplasia. Mol. Cancer Res..

[85] Ng E.K., Tsang W.P., Ng S.S.M., Jin H.C., Yu J., Li J.J., Rocken C., Ebert M.P.A., Kwok T.T., Sung J.J.Y. (2009). MicroRNA-143 targets DNA methyltransferases 3A in colorectal cancer. Br. J. Cancer.

[86] Bandrés E., Cubedo E., Agirre X., Malumbres R., Zarate R., Ramirez N., Abajo A., Navarro A., Moreno I., Monzo M. (2006). Identification by Real-time PCR of 13 mature microRNAs differentially expressed in colorectal cancer and non-tumoral tissues. Mol. Cancer.

[87] Fabbri M., Paone A., Calore F., Galli R., Gaudio E., Santhanam R., Lovat F., Fadda P., Mao C., Nuovo G.J. (2012). MicroRNAs bind to Toll-like receptors to induce prometastatic inflammatory response. Proc. Natl. Acad. Sci. USA.

[88] Schepeler T., Reinert J.T., Ostenfeld M.S., Christensen L.L., Silahtaroglu A.N., Dyrskjot L., Wiuf C., Sorensen F.J., Kruhoffer M., Laurberg S. (2008). Diagnostic and prognostic microRNAs in stage II colon cancer. Cancer Res..

[89] Milanesi E., Dobre M., Bucuroiu A.I., Herlea V., Manuc T.E., Salvi E., De Petro G., Manuc M., Becheanu G. (2020). miRNAs-Based Molecular Signature for KRAS Mutated and Wild Type Colorectal Cancer: An Explorative Study. J. Immunol. Res..

[90] Gasparello J., Papi C., Allegretti M., Giordani E., Carboni F., Zazza S., Pescarmona E., Romania P., Giacomini P., Scapoli C. (2020). A distinctive microRNA (miRNA) SIgature in the Blood of Colorectal Cancer (CRC) Patients at Surgery. Cancers.

[91] Caramés C., Cristóbal I., Moreno V., del Puerto L., Moreno I., Rodriguez M., Marín J.P., Correa A.V., Hernández R., Zenzola V. (2015). MicroRNA-21 predicts response to preoperative chemoradiotherapy in locally advanced rectal cancer. Int. J. Colorectal Dis..

[92] Kjersem J.B., Ikdahl T., Lingjaerde O.C., Guren T., Tveit K.M., Kure E.H. (2014). Plasma microRNAs predicting clinical outcome in metastatic colorectal cancer patients receiving first-line oxaliplatin-based treatment. Mol. Oncol..

[93] Lee Y., Kim S.J., Choo J., Heo J., Yoo J.W., Jung Y., Ree S.H., Im E. (2020). miR-23a-3p is a Key Regulator of IL-17C-Induced Tumor Angiogenesis in Colorectal Cancer. Cells.

[94] Amerizadeh F., Khazaei M., Maftouh M., Mardani R., Bahrami A. (2018). miRNA Targeting Angiogenesis as a Potential Therapeutic Approach in the Treatment of Colorectal Cancers. Curr. Pharm. Des..

[95] Ruzzo A., Graziano F., Vincenzi B., Canestrari E., Perrone G., Galluccio N., Catalano V., Loupakis F., Rabitti C., Santini D. (2012). High let-7° microRNA levels in KRAS-mutated colorectal carcinomas may rescue anti-EGFR therapy effects in patients with chemotherapyrefractory metastatic disease. Oncologist.

[96] Valeri N., Gasparini P., Braconi C., Paone A., Lovat F., Fabbri M., Sumani K.M., Alder H., Amadori D., Patel T. (2010). Croce CM. MicroRNA-21 induces resistance to 5-fluorouracil by down-regulating human DNA MutS homolog 2 (hMSH2). Proc. Natl. Acad. Sci. USA.

[97] Wagenaar T.R., Zabludoff S., Ahn S.M., Allerson C., Arlt H., Baffa R., Cao H., Davis S., Garcia-Echeverria C., Gaur R. (2015). Anti-miR-21 Suppresses Hepatocellular Carcinoma Growth via Broad Transcriptional Network Deregulation. Mol. Cancer Res..

[98] Nishida N., Yamashita S., Mimori K., Sudo T., Tanaka F., Shibata K., Yamamoto H., Ishii H., Doki Y., Mori M. (2012). MicroRNA-10b is a prognostic indicator in colorectal cancer and confers resistance to the chemotherapeutic agent 5-fluorouracil in colorectal cancer cells. Ann. Surg. Oncol..

[99] Poorebrahim M., Sadeghi S., Ghanbarian M., Kalhor H., Mehrtash A., Teimoori-Toolabi L. (2020). Identification of candidate genes and miRNAs for sensitizing resistant colorectal cancer cells to oxaliplatin and irinotecan. Cancer Chemother. Pharmacol..

[100] Fernandez-Medarde A., Santos E. (2011). Ras in cancer and developmental diseases. Genes Cancer.

[101] Ciombor K.K., Bekaii-Saab T. (2018). A Comprehensive Review of Sequencing and Combination Strategies of Targeted Agents in Metastatic Colorectal Cancer. Oncologist.

[102] Cox A.D., Fesik S.W., Kimmelman A.C., Luo J., Der C.J. (2014). Drugging the undruggable RAS: Mission possible?. Nat. Rev. Drug. Discov..

[103] Irahara N., Baba Y., Nosho K., Shima K., Yan L., Dias-Santagata D., Iafrate A.J., Fuchs C.S., Haigis K.M., Ogino S. (2010). NRAS mutations are rare in colorectal cancer. Diagn. Mol. Pathol..

[104] He Z., Thorrez L., Siegfried G., Meulemans S., Evrard S., Tejpar S., Khatib A.-M., Creemers J.W.M. (2020). The proprotein convertase furin is a pro-oncogenic driver in KRAS and BRAF driven colorectal cancer. Oncogene.

[105] Yokota T., Shibata N., Ura T., Takahari D., Shitara K., Muro K., Yatabe Y. (2010). Cycleave polymerase chain reaction method is practically applicable for V-Ki-ras2 Kirsten rat sarcoma viral oncogene homolog (KRAS)/V-raf murine sarcoma viral oncogene homolog B1 (BRAF) genotyping in colorectal cancer. Transl. Res..

[106] Smakman N., Borel Rinkes I.H.M., Voest E.E., Kranenburg O. (2005). Control of colorectal metastasis formation by K-Ras. Biochim. Biophys. Acta.

[107] Allgayer H., Wang H., Shirasawa S., Sasazuki T., Boyd D. (1999). Targeted disruption of the K-ras oncogene in an invasive colon cancer cell line down-regulates urokinase receptor expression and plasminogen-dependent proteolysis. Br. J. Cancer.

[108] Di Mauro C., Pesapane A., Formisano L., Rosa R., D’amato V., Ciciola P., Servetto A., Marciano R., Orsini R.C., Monteleone F. (2017). Urokinase-type plasminogen acrivator receptor (uPAR) expression enhances invasion and metastasis in RAS mutated tumors. Sci. Rep..

[109] Yamamoto H., Itoh F., Senota A., Adachi Y., Yoshimoto M., Endoh T., Hinoda Y., Yachi A., Imai K. (1995). Expression of matrix metalloproteinase matrilysin (MMP-7) was induced by activated Ki-ras via AP-1 activation in SW1417 colon cancer cells. J. Clin. Lab. Anal..

[110] Samowitz W.S., Curtin K., Schaffer D., Robertson M., Leppert M., Slattery M.L. (2000). Relationship of Ki-ras mutations in colon cancers to tumor location, stage, and survival: A population-based study. Cancer Epidemiol. Biomark. Prev..

[111] Bazan V., Migliavacca M., Zanna I., Tubiolo C., Grassi N., Latteri M.A., La Farina M., Albanese I., Dardanoni G., Salerno S. (2002). Specific codon 13 K-ras mutations are predictive of clinical outcome in colorectal cancer patients, whereas codon 12 K-ras mutations are associated with mucinous histotype. Ann. Oncol..

[112] Jiang Y., Kimchi E.T., Staveley-O’Carroll K.F., Cheng H., Ajani J.A. (2009). Assessment of K-ras mutation: A step toward personalized medicine for patients with colorectal cancer. Cancer.

[113] Binefa G., Rodriguez-Moranta F., Teule A., Medina-Hayas M. (2014). Colorectal cancer: From prevention to personalized medicine. World J. Gastroenterol..

[114] Rose J.S., Serna D.S., Martin L.K., Li X., Weatherby L.M., Abdel-Misih S., Zhao W., Bekaii-Saab T. (2012). Influence of KRAS mutation status in metachronous and synchronous metastatic colorectal adenocarcinoma. Cancer.

[115] Charlton M.E., Kahl A.R., Greenbaum A.A., Karlitz J.J., Lin C., Lynch C.F., Chen V.W. (2017). KRAS Testing, Tumor Location, and Survival in Patients with Stage IV Colorectal Cancer: SEER 2010–2013. J. Natl. Compr. Cancer Netw..

[116] Tsilimigras D.I., Ntanasis-Stathopoulos I., Bagante F., Moris D., Cloyd J., Spartalis E., Pawlik T.M. (2018). Clinical significance and prognostic relevance of KRAS, BRAF, PI3K and TP53 genetic mutation analysis for resectable and unresectable colorectal liver metastases: A systematic review of the current evidence. Surg. Oncol..

[117] Denbo J.W., Yamashita S., Passot G., Egger M., Chun Y.S., Kopetz S.E., Maru D., Brudvik K.W., Wei S.H., Conrad C. (2017). RAS Mutation Is Associated with Decreased Survival in Patients Undergoing Repeat Hepatectomy for Colorectal Liver Metastases. J. Gastrointest. Surg..

[118] Lo Nigro C., Ricci V., Vivenza D., Granetto C., Fabozzi T., Miraglio E., Merlano M.C. (2016). Prognostic and predictive biomarkers in metastatic colorectal cancer anti-EGFR therapy. World J. Gastroenterol..

[119] Ulz P., Heitzer E., Geigl J.B., Speicher M.R. (2017). Patient monitoring through liquid biopsies using circulating tumor DNA. Int. J. Cancer.

[120] Oellerich M., Schutz E., Beck J., Walson P.D. (2019). Circulating Cell-Free DNA-Diagnostic and Prognostic Applications in Personalized Cancer Therapy. Ther. Drug. Monit..

[121] Furuki H., Yamada T., Takahashi G., Iwai T., Koizumi M., Shinji S., Yokoyama Y., Takeda K., Taniai N., Uchida E. (2018). Evaluation of liquid biopsies for detection of emerging mutated genes in metastatic colorectal cancer. Eur. J. Surg. Oncol..

[122] Fearon E.R. (2011). Molecular genetics of colorectal cancer. Annu. Rev. Pathol..

[123] Sepulveda A.R., Hamilton S.R., Allegra C.J., Grody W., Cushman-Vokoun A.M., Funkhouser W.K., Kopetz S.E., Lieu C., Lindor N.M., Minsky B.D. (2017). Molecular Biomarkers for the Evaluation of Colorectal Cancer: Guideline Summary From the American Society for Clinical Pathology, College of American Pathologists, Association for Molecular Pathology, and American Society of Clinical Oncology. J. Oncol. Pract..

[124] Sanz-Garcia E., Argiles G., Elez E., Tabernero J. (2017). BRAF mutant colorectal cancer: Prognosis, treatment, and new perspectives. Ann. Oncol..

[125] Barras D. (2015). BRAF Mutation in Colorectal Cancer: An Update. Biomark. Cancer.

[126] Matallanas D., Birtwistle M., Romano D., Zebisch A., Rauch J., von Kriegsheim A., Kolch W. (2011). Raf family kinases: Old dogs have learned new tricks. Genes Cancer.

[127] Wan P.T.C., Garnett M.J., Roe S.M., Lee S., Niculescu-Duvaz D., Good V.M., Jones C.M., Marshall C.J., Springer C.J., Barford D. (2004). Mechanism of activation of the RAF-ERK signaling pathway by oncogenic mutations of B-RAF. Cell.

[128] Davies H., Bignell G.R., Cox C., Stephens P., Edkins S., Clegg S., Teague J., Woffendin H., Garnett M.J., Bottomley W. (2002). Mutations of the BRAF gene in human cancer. Nature.

[129] Cohen R., Pudlarz T., Delattre J.F., Colle R., André T. (2020). Molecular Targets for the Treatement of Metastatic Colorectal Cancer. Cancers.

[130] Chen D., Huang J.F., Liu K., Zhang L.Q., Yang Z., Chuai Z.R., Wang Y.X., Shi D.C., Huang Q., Fu W.L. (2014). BRAFV600E mutation and its association with clinicopathological features of colorectal cancer: A systematic review and meta-analysis. PLoS ONE.

[131] Clarke C.N., Kopetz E.S. (2015). BRAF mutant colorectal cancer as a distinct subset of colorectal cancer: Clinical characteristics, clinical behavior, and response to targeted therapies. J. Gastrointest. Oncol..

[132] Ahronian L.G., Sennott E.M., Van Allen E.M., Wagle N., Kwak E.L., Faris J.E., Godfrey J.T., Nishimura K., Lynch K.D., Mermel C.H. (2015). Clinical Acquired Resistance to RAF Inhibitor Combinations in BRAF-Mutant Colorectal Cancer through MAPK Pathway Alterations. Cancer Discov..

[133] Tie J., Desai J. (2015). Targeting BRAF mutant metastatic colorectal cancer: Clinical implications and emerging therapeutic strategies. Target Oncol..

[134] Clancy C., Burke J.P., Kalady M.F., Coffey J.C. (2013). BRAF mutation is associated with distinct clinicopathological characteristics in colorectal cancer: A systematic review and meta-analysis. Colorectal Dis..

[135] Missiaglia E., Jacobs B., D’Ario G., Di Narzo A.F., Soneson C., Budinska E., Popovici V., Vecchione L., Gerster S., Yan P. (2014). Distal and proximal colon cancers differ in terms of molecular, pathological, and clinical features. Ann. Oncol..

[136] Weisenberger D.J., Siegmund K.D., Campan M., Young J., Long T.I., Faasse M.A., Kang G.H., Widschwendter M., Weener D., Buchanan D. (2006). CpG island methylator phenotype underlies sporadic microsatellite instability and is tightly associated with BRAF mutation in colorectal cancer. Nat. Genet..

[137] Tie J., Gibbs P., Lipton L., Christie M., Jorissen R.N., Burgess A.W., Croxford M., Jones I., Langland R., Kosmider S. (2011). Optimizing targeted therapeutic development: Analysis of a colorectal cancer patient population with the BRAF(V600E) mutation. Int. J. Cancer.

[138] Lochhead P., Kuchiba A., Imamura Y., Liao X., Yamauchi M., Nishihara R., Qian Z.R., Morikawa T., Shen J., Meyerhardt J.A. (2013). Microsatellite instability and BRAF mutation testing in colorectal cancer prognostication. J. Natl. Cancer Inst..

[139] Landau M.S., Kuan S.F., Chiosea S., Pai R.K. (2014). BRAF-mutated microsatellite stable colorectal carcinoma: An aggressive adenocarcinoma with reduced CDX2 and increased cytokeratin 7 immunohistochemical expression. Hum. Pathol..

[140] Dankner M., Rose A.A.N., Rajkumar S., Siegel P.M., Watson I.R. (2018). Classifying BRAF alterations in cancer: New rational therapeutic strategies for actionable mutations. Oncogene.

[141] De Roock W., Claes B., Bernasconi D., De Schutter J., Biesmans B., Fountzilas G., Kalogeras K.T., Kotoula V., Papamichael D., Laurent-Puig P. (2010). Effects of KRAS, BRAF, NRAS, and PIK3CA mutations on the efficacy of cetuximab plus chemotherapy in chemotherapy-refractory metastatic colorectal cancer: A retrospective consortium analysis. Lancet Oncol..

[142] Fanelli G.N., Dal Pozzo C.A., Depetris I., Schirripa M., Brignola S., Biason P., Balistreri M., Dal Santo L., Lonardi S., Munari G. (2020). The heterogeneous clinical and pathological landscapes of metastatic Braf-mutated colorectal cancer. Cancer Cell Int..

[143] Ogino S., Shima K., Meyerhardt J.A., McCleary J., Ng K., Hollis D., Saltz L.B., Mayer R.J., Schaefer P., Whittom R. (2012). Predictive and prognostic roles of BRAF mutation in stage III colon cancer: Results from intergroup trial CALGB 89803. Clin. Cancer Res..

[144] Loupakis F., Intini R., Cremolini C., Orlandi A., Sartore-Bianchi A., Pietrantonio F., Pella N., Spallanzani A., Dell’Aquila E., Scartozzi M. (2019). A validated prognostic classifier for (V600E)BRAF-mutated metastatic colorectal cancer: The ‘BRAF BeCool’ study. Eur. J. Cancer.

[145] Di Nicolantonio F., Martini M., Molinari F., Sartore-Bianchi A., Arena S., Saletti P., De Dosso S., Mazzucchelli L., Frattini M., Siena S. (2008). Wild-type BRAF is required for response to panitumumab or cetuximab in metastatic colorectal cancer. J. Clin. Oncol..

[146] Modest D.P., Martens U.M., Riera-Knorrenschild J., Greeve J., Florschutz A., Wessendorf S., Ettrich T., Kanzler S., Norenberg D., Ricke J. (2019). FOLFOXIRI Plus Panitumumab As First-Line Treatment of RAS Wild-Type Metastatic Colorectal Cancer: The Randomized, Open-Label, Phase II VOLFI Study (AIO KRK0109). J. Clin. Oncol..

[147] Flaherty K.T., Puzanov I., Kim K.B., Ribas A., McArthur G.A., Sosman J.A., O’Dwye P.J., Lee R.J., Grippo J.F., Nolop K. (2010). Inhibition of mutated, activated BRAF in metastatic melanoma. N. Engl. J. Med..

[148] Prahallad A., Sun C., Huang S., Di Nicolantonio F., Salazar R., Zecchin D., Beijersbergen R.L., Bardelli A., Bernards R. (2012). Unresponsiveness of colon cancer to BRAF(V600E) inhibition through feedback activation of EGFR. Nature.

[149] Kopetz S., Grothey A., Yaeger R., Van Cutsem E., Desai J., Yoshino T., Wasan H., Ciardiello F., Loupakis F., Sang Hong Y. (2019). Encorafenib, Binimetinib, and Cetuximab in BRAF V600E-Mutated Colorectal Cancer. N. Engl. J. Med..

